# Generation of a Recombinant Porcine Reproductive and Respiratory Syndrome Virus Stably Expressing Two Marker Genes

**DOI:** 10.3389/fvets.2020.548282

**Published:** 2020-10-22

**Authors:** Hao Wang, Xin Xie, Wei He, Yuxu Wang, Tongwei Ren, Kang Ouyang, Ying Chen, Weijian Huang, Zuzhang Wei

**Affiliations:** Laboratory of Animal Infectious Diseases and Molecular Immunology, College of Animal Science and Technology, Guangxi University, Nanning, China

**Keywords:** PRRSV, expression vector, marker genes, genetic stable, insertion

## Abstract

Porcine reproductive and respiratory syndrome virus (PRRSV) has been used as a gene expression vector in the development of vaccines. Most of these recombinant PRRSV vectors express only a single foreign gene through either an internal insertion in the hypervariable region of nsp2 or expression cassette and some of these recombinant vectors are genetically unstable. Here, we combined internal insertion in nsp2 and expression cassette methods to generate a novel recombinant PRRSV stably expressing the red fluorescence protein (RFP) and the green fluorescence protein (GFP) genes. Biological characteristic analysis of the recombinant PRRSV carrying the two marker genes, rGX-RFP-GFP, showed that it displayed similar growth kinetics and yet it yielded less infectious viruses when compared to the parental virus rGXAM. Co-expression of both the RFP and GFP was observed using confocal fluorescence microscopy when the rGX-RFP-GFP viruses infected MARC-145 cells. Furthermore, the PRRSV-based two-marker gene expression vector is genetically stable during 20 serial passages in MARC-145 cells. These data demonstrate that it is possible to express two interested immunogens from a single PRRSV vector.

## Introduction

Porcine reproductive and respiratory syndrome virus (PRRSV) is an enveloped, non-segmented, positively-stranded RNA virus and has been classified in the genus Porartevirus, family *Arteriviridae* and order *Nidovirales* (1). The virus is the causative agent of porcine reproductive and respiratory syndrome (PRRS) which could lead to respiratory distress in piglets and reproductive failure in pregnant sows (2). PRRSV can be classified into PRRSV-1 and PRRSV-2 genotypes. The PRRSV genome consists of ~15.5 kb and consists of a 5′-untranslated region (UTR), a 3′-UTR and at least nine open reading frames (ORFs). The 5′ two-thirds of the genome encodes two polyproteins (pp1a and pp1ab) which are processed by viral proteases to at least 16 replicase-related non-structural proteins (nsps) and these play a key role in viral replication and transcription. The other one-third of the genome mostly encodes structural proteins including glycoprotein (GP) 2a, 2b, GP3, ORF5a protein, GP5, M, and N (3, 4). All the structural proteins of PRRSV are essential for virus replication (5–7).

The availability of reverse genetics systems for PRRSV has created new perspectives for the use of recombinant PRRSV as expression vectors (8–14). Nsp2 is one of the most variable proteins in PRRSV. Natural spontaneous amino acids deletions and insertions were found in the hypervariable region (HVR) of nsp2 (15–19). The HVR of nsp2 can also be engineered for the insertion of foreign genes or deletion of specific individual immune dominant epitopes in order to develop recombinant marker PRRSV vaccines (8, 17, 20–24). However, fusion of the foreign genes within the HVR of nsp2 in most recombinant viruses does not always maintain stability of the resultant recombinant viruses or sometimes there is a loss of biological activity of the target genes after serial passages in MARC-145 cells (8, 22, 23). Another additional strategy for the expression of a foreign gene was achieved through insertion of an extra independent transcription unit to produce an extra sub-genomic (sg) mRNA (25, 26). The intergenic regions between ORF1b and 2 or ORF7 and 3′UTR, which are separated by one nucleotide or without overlapping, respectively, were found to be ideal sites for the insertion of expression cassettes so as to express the foreign genes (25, 26). The inserted independent transcription unit which is responsible for transcription of the foreign gene is regulated by the genomic transcriptional regulatory sequence (TRS) upstream of the inserted foreign sequences. In this case, a synthetically copied TRS with flanking sequences was placed after the inserted gene to regulate the synthesis of sub-genomic (sg) mRNA of the downstream viral structural protein (25, 26). Most recombinant PRRSVs expressing foreign genes including marker genes, immunogenic genes of viruses and cytokine genes between ORF1 and 2 or ORF7 and 3′ UTR, show genetic stability and have the biological activity of the foreign genes during serial passages of recombinant viruses both *in vitro* and *in vivo* (27–32).

In this study, for the first time, we combined the internal insertion in nsp2 and independent transcription unit approaches to generate a novel PRRSV-based recombinant virus expressing both RFP and GFP genes. The recombinant viruses maintain similar growth characteristics with the parental virus and are genetically stable in MARC-145 cells.

## Materials and Methods

### Cells, Viruses, and Plasmids

MARC-145 and BHK-21 (ATCC, CCL10) cells were cultured as described previously (10). The infectious PRRSV-2 cDNA clone pGXAM was derived from a cell adapted PRRSV strain, GXNN1396-P96 (Genbank accession no. MN660068) (33).

### Plasmid Construction

**Figure 1:**
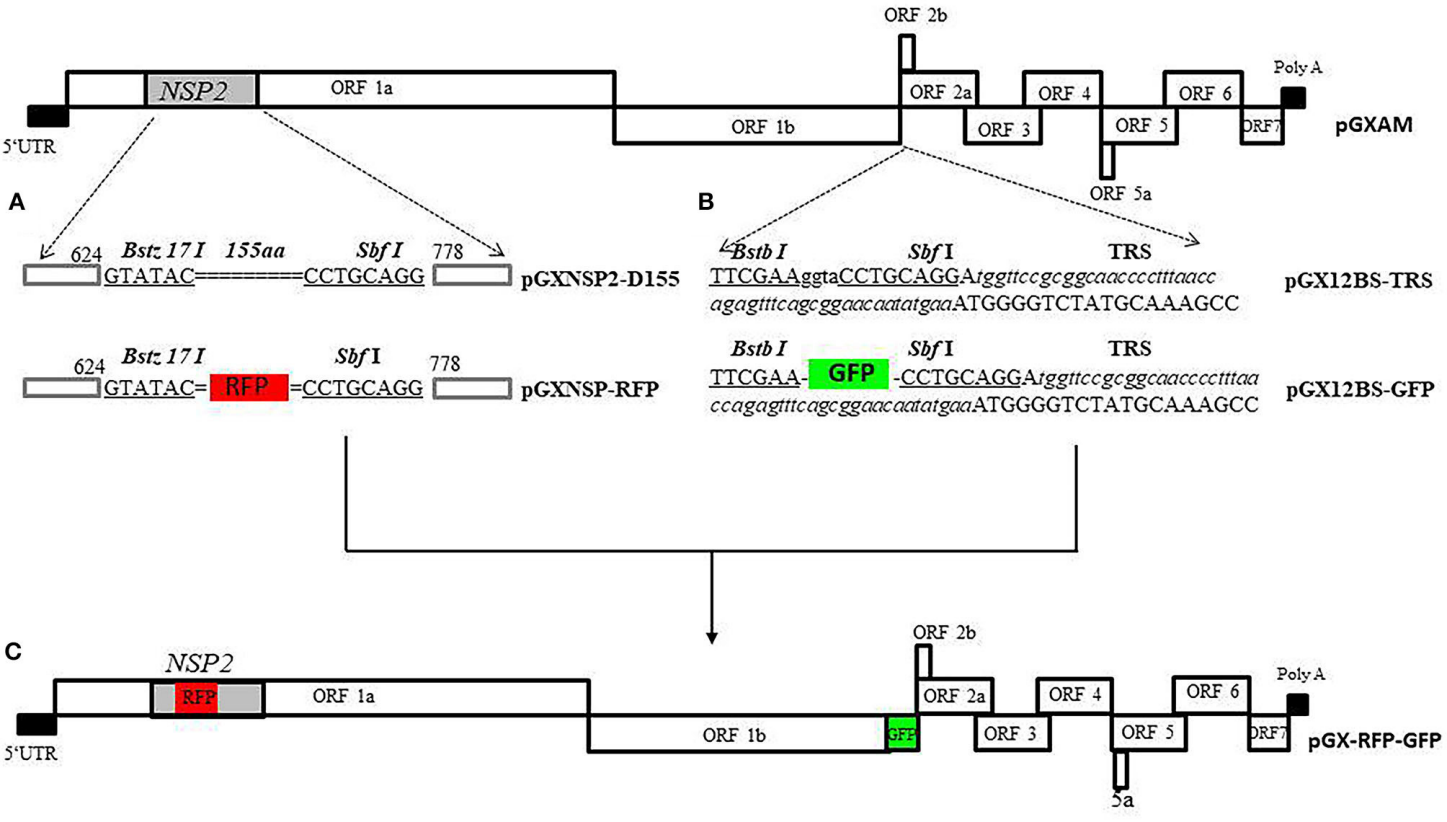
The overall scheme of the cloning strategy for the full-length PRRSV cDNA clone carrying RFP and GFP. **(A)** Construction a recombinant PRRSV virus expressing RFP in nsp2. Site-directed mutagenesis was accomplished by splicing the overlapping extensions to generate the mutant plasmid (pGX-NSP2-D465) carrying the 155-aa deletion in nsp2. The double dotted line represents the position of the deletion. The two introduced unique restriction enzyme sites, Bstz 17 I and SbfI, are indicated by boldface type and underlined. The RFP gene is boxed. **(B)** Construction of recombinant PRRSV with GFP gene inserted in the ORF1/2 junction region. Recombinant clone, pGXBS-TRS, serves as a PRRSV expression cassette for foreign gene insertion. The two introduced unique restriction enzyme sites, BstB I and SbfI, are indicated by boldface type and underlined. The introduced body TRS with flanking sequences are underlined with italic type. **(C)** The full-length PRRSV cDNA clone carrying RFP and GFP.

Figure 1 shows the cloning strategy for the infectious PRRSV-2 cDNA clone pGXAM carrying the RFP and GFP genes. For the convenience of genetic manipulations, a fragment was amplified by PCR with forward primer, JX451F, and reverse primer, JX6463R, using pGXAM as template. The PCR product was then cloned into the Zero Blunt® PCR vector, resulting in a shuttle plasmid (pTOPO-P2). To generate the construct carrying the 155-aa deletion in HV of nsp2, the intermediate PCR products were amplified using mutagenic primers (Nsp2 D155 BstZ17IF and Nsp2 D155 SbfI R) and flanking primers (GX2350F and GX3921R) and the resulting two segments were then spliced in the second round of fusion PCR. The final PCR products were digested with Afe I and Bbs I and ligated into similarly digested pTOPO-P2. The fragments bearing the 155-aa deletion and introduced restriction enzymes (BstZ17IF and SbfI) were digested with SpeI and Afl II and ligated into similarly digested corresponding regions of pGXAM, resulting in the recombinant clone, pGX-NSP2-D465. To construct a recombinant clone carrying RFP, the RFP gene was amplified using primers RFP BstZ17I F and RFP SbfI R. The resulting PCR products were inserted between BstZ17I F and SbfI R in pGX-NSP2-D465 through homologous recombination using a one-step cloning kit (Vazyme), resulting in the recombinant clone, pGX-NSP2-RFP.

In order to generate the expression cassette in ORF1b and ORF2a, an upstream fragment was amplified from t pGXAM with forward primer, GF11706, and reverse primer, 12BSR, containing the BstB I and SbfI restriction enzyme sites. A downstream fragment was amplified from the backbone of the pGXAM infectious clone with forward primer, 12BSF containing the BstB I and SbfI restriction enzyme sites, and reverse primer, JX14402Mlu I R. The resulting upstream and downstream fragments were then spliced in the second round of fusion PCR using GF11706 and JX14402 Mlu I R. The resulting PCR products were cloned into the Zero Blunt® PCR vector, resulting a shuttle plasmid (pTOPO-12BS). A fragment was amplified from pTOPO-12BS with forward primer, SbfI-TRS containing the SbfI restriction enzyme site and a synthetic TRS and reverse primer, JX14402MluI R. The fragments bearing the introduced restriction enzyme Sbf I site and the TRS sequence and the shuttle plasmid pTOPO-12BS were digested with SbfI and Mlu I and then ligated to generate a shuttle plasmid (pTOPO-12BS-TRS). pTOPO-12BS-TRS and pGXAM were digested with Asc I and Mlu I, and then ligated by T4 DNA ligase in order to construct a recombinant infectious clone pGXBS-TRS. The pGXBS-TRS contained two restriction enzyme sites, BstB I and Sbf I, and a synthetic TRS that would allow for the insertion of an expression cassette. The GFP gene was amplified using primers GFP BstBI F and GFP SbfI R. The resulting PCR products which were digested with the restriction enzymes, Sbf I and BstB I, were inserted between BstB I and Sbf I in pGXBS-TRS which was previously digested with similar restriction enzymes and this resulted in the recombinant clone, pGXBS-TRS-GFP. To construct a recombinant PRRSV clone containing the two marker genes, pGXBS-TRS-GFP was digested with Asc I and Mlu I, and then ligated into similarly digested pGX-NSP2-RFP, resulting in the recombinant clone, pGX-RFP-GFP, which carried the two fluorescent marker genes. All the primers used in this study are listed in Supplementary Table 1.

### DNA Transfection and Recovery of Recombinant PRRS

Plasmids were purified using a QIAprep Spin Miniprep kit (Qiagen, Hilden, Germany). BHK-21 cells grown in 6-well cell culture plates were transfected with 1 μg of purified plasmids using Fugene HD Transfection Reagent (Roche, USA) according to the manufacturer's instructions. The transfection mixture was discarded at 6 h post-transfection (hpt) and cells were further incubated in fresh EMEM containing 2% FBS for 24 h. For virus rescue, 100 μL supernatant of transfected BHK-21 cells was harvested and used to inoculate fresh MARC-145 cells and incubated for 1 h. After washing with PBS, MARC-145 cells were then incubated with 3 mL of EMEM containing 2% FBS for 4 days. Recovered viruses were designated as the primary passage (P0). The P0 viruses were used for serial 20 passages (P1–P20) in MARC-145 cells using a 10^2^ dilution at each passage.

### Indirect Immunofluorescence Assay (IFA)

The expression of viral N proteins in PRRSV infected MARC-145 cells were tested by indirect immunofluorescent assay (IFA) as described in our previous study (10). Briefly, the monolayers of MARC-145 cells were infected with the recombinant or parental viruses. At 48 h post-infection (hpi), MARC-145 cells were fixed using cold methanol for 15 min at room temperature and blocked with 3% BSA (fraction V bovine serum albumin) (Roche, Mannheim, Germany) for 30 min. The fixed cells were then incubated with a monoclonal antibody (mAb) against PRRSV N protein (SR30-A) (Rural Technologies Inc., Brookings, SD, USA) for 2 h at 37°C. After washing with PBS, Alexa Fluor 488-conjugated goat anti- mouse IgG or Alexa Fluor 568-conjugated goat anti-mouse IgG (Thermo Fisher Scientific) (1:1000) as the secondary antibody was incubated for 60 min at 37°C. The cells were washed five times with PBS. Finally, images were captured using an Olympus inverted fluorescence microscope fitted with a CCD camera.

### Multi-Step Growth Curve

To assess the growth curve, MARC-145 cells cultured in six-well plates were inoculated with the recombinant viruses and parental viruses at 0.1 multiplicity of infection (MOI). 200 μL of culture supernatant was harvested at the indicated times (12, 24, 48, 72, 96, and 120 hpi), and frozen at −80°C until used. The viral titers (log10 TCID_50_/mL) of harvested supernatants for each time point were assayed in MARC-145 cells and calculated according to the Reed & Muench method (34). Each experiment was repeated independently three times and the SEM was calculated.

### RT-PCR

The genetic stability of insertion genes in PRRSV genomic RNA of recombinant viruses was analyzed by sequencing of the RT-PCR products. Viral genomic RNA was isolated from the viral supernatants of cells infected with the P3, P5, P10, P15, and P20 viruses using a QIAamp Viral RNA Mini Kit (QIAgen, Hilden, Germany), according to the manufacturer's protocol. Viral genomic RNA was used for synthesis of cDNA using avian myeloblastosis virus (AMV) reverse transcriptase (TaKaRa, Dalian, China). The fragments containing the deletion regions or the insertion genes were amplified using the respected specific primers which are listed in Supplementary Table 1. The PCR products were purified using a TIANgel Mini Purification Kit (TIANGEN) and then subjected to nucleotide sequencing.

### Statistical Analysis

Virus titers are presented as mean ± SEM. Statistical analysis of virus titers of harvested supernatants for each time point was performed using two-way ANOVA in the generalized linear model (GLM) of SPSS 12.0 for Windows (SPSS Inc., Chicago, IL, USA). Before performing any statistical tests, Bartlett and Shapiro-Wilk tests, respectively, were used to check the homogeneity of variance and normality distribution of the data. Values of *P* <0.05 was used as the criterion for determining the significant difference.

## Results

### Construction and Characterization of the PRRSV Virus Expressing RFP in nsp2

To express the reporter gene (RFP) in nsp2, a recombinant clone (pGX-NSP2-D465) carrying a 465 nt deletion in the HV nsp2 that previously occurred naturally in a PRRSV isolate, GXNN2465 (Genbank no.MN816496), was constructed. In order to insert the reporter gene into nsp2, two restriction enzymes (BstZ17IF and SbfI) were introduced in the deleted region. The RFP gene was inserted into the pGX-NSP2-D465 at the BstZ17IF and SbfI sites to generate recombinant clone pGX-NSP2-RFP, as shown in Figure 1.

**Figure 2:**
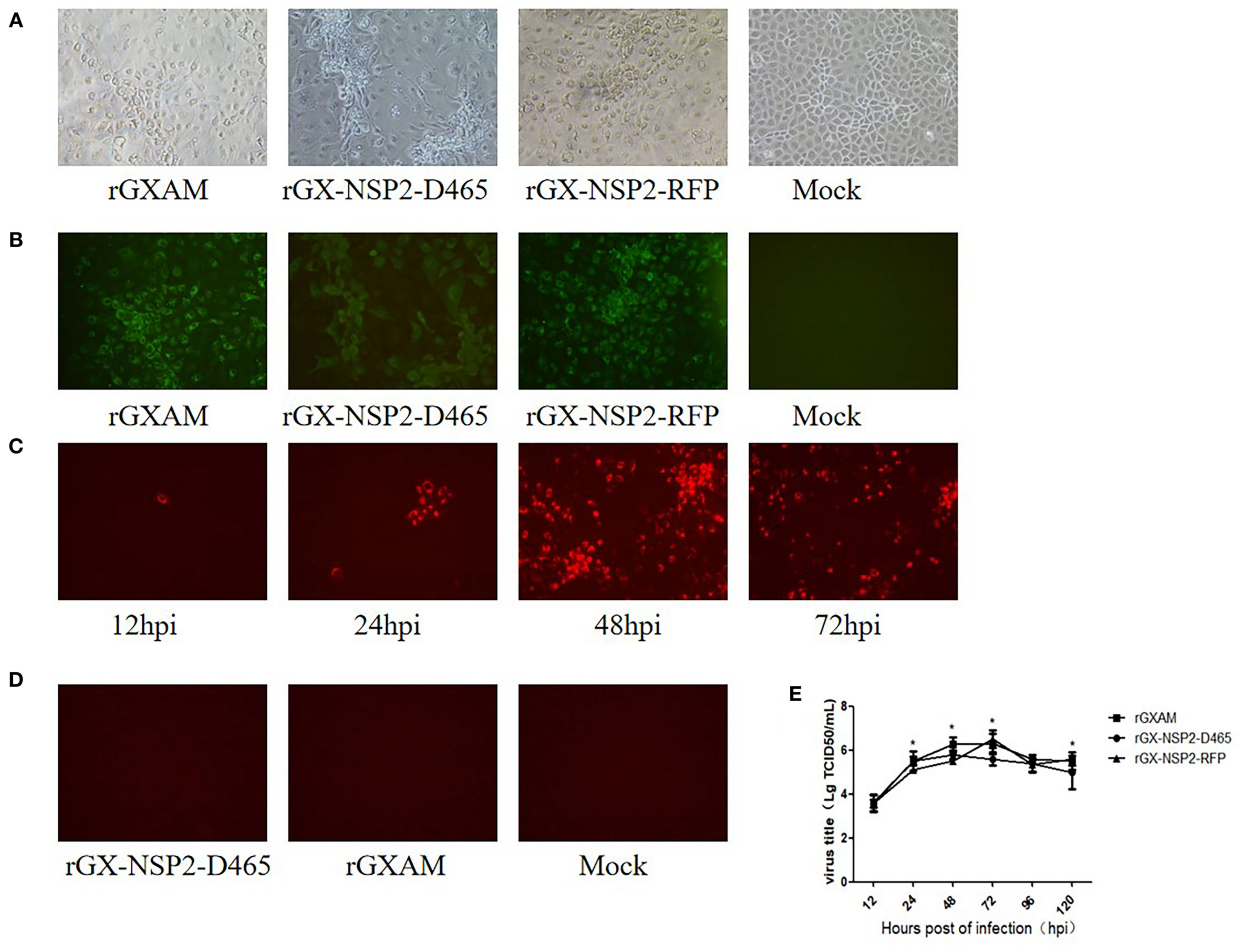
Recovery of PRRSV recombinant viruses with the 155-aa deletion and insertion GFP gene in nsp2. **(A)** CPE of recombinant viruses and parental PRRSV infected MARC-145 cells. Mock infected and recombinant virus infected MARC-145 cells were observed 72 h post-transfection. **(B)** Detection of the expression of N protein by using IFA. The infected MARC-145 cells were fixed and stained using a monoclonal antibody against the PRRSV N protein and anti-mouse secondary antibody labeled with Alexa Fluor 488. Images were taken at ×200 magnification. **(C)** MARC-145 cells were seeded in 6-well plates and infected with rGX-NSP2-RFP at a MOI of 0.1. At different times after infection, live cells were imaged with a fluorescence microscope. **(D)** MARC-145 cells were seeded in 6-well plates and infected with rGX-NSP2-D465 or rGXAM. At 72 h after infection, live cells were imaged with a fluorescence microscope. **(E)** Comparison of the growth kinetics of recombinant and parental viruses. The recombinant and the parental viruses were used to infect monolayers of MARC-cells. Subsequently, 200 μL of the supernatant was collected at the time indicated. The viral titers were determined as TCID_50_ and the values obtained were the means of using three independent experiments. Virus titers are expressed as mean ± SEM. *Indicates significant differences between the recombinant viruses and the parental virus.

To test whether the recombinant clones are infectious, pGX-NSP2-D465, pGX-NSP2-RFP, and pGXAM were transfected into BHK-21 cells. At 30 hpt, fresh MARC-145 cells were inoculated with the supernatants of the transfected BHK-21 cells for recovery of the recombinant viruses. Cytopathic effects (CPE) were developed in MARC-145 cells at 72 hpi, as shown in Figure 2A. IFA showed that PRRSV N protein expression was evident in these MARC-145 cells at 72 hpi (Figure 2B). The RFP positive cells were observed in rGX-NSP2-RFP infected cells as early as 12 hpi. As infection progressed, there were more RFP positive cells at 72 and 96 hpi which indicated the spread of virus infection (Figure 2C). As expected, rGX-NSP2-D465 and rGXAM-infected cells did not show any red fluorescence (Figure 2D). To further characterize the recombinant viruses, their multi-step growth kinetics were determined in virus infected MARC-145 cells. The results showed that parental and recombinant viruses displayed similar growth behavior. The overall yields were not significantly different between parental virus and recombinant viruses (Figure 2E). Overall, the results show that the reporter virus is replication-competent and infectious.

### Construction and Characterization of the PRRSV Virus Expressing GFP in a Transcript Unit Inserted Between ORF1 and ORF2

Two restriction enzyme sites, Sbf I and BstB I, were introduced into the region between ORF1b and ORF2a of pGXAM to provide the facility for the insertion of any foreign genes. The foreign gene could be transcribed into a novel sg mRNA guided by TRS2 which would have originally been responsible for ORF2a transcription. The transcription sg mRNA for ORF2a is compensated for by insertion of a synthetic body TRS with flanking sequences immediately after the Sbf I site. The resulting construct was designated pGXBS-TRS (Figure 1). Subsequently, the GFP gene was cloned into the pGXBS-TRS by using the two restriction enzyme sites, Sbf I and BstB I. The resulting construct was designated pGXBSTRS-GFP.

**Figure 3:**
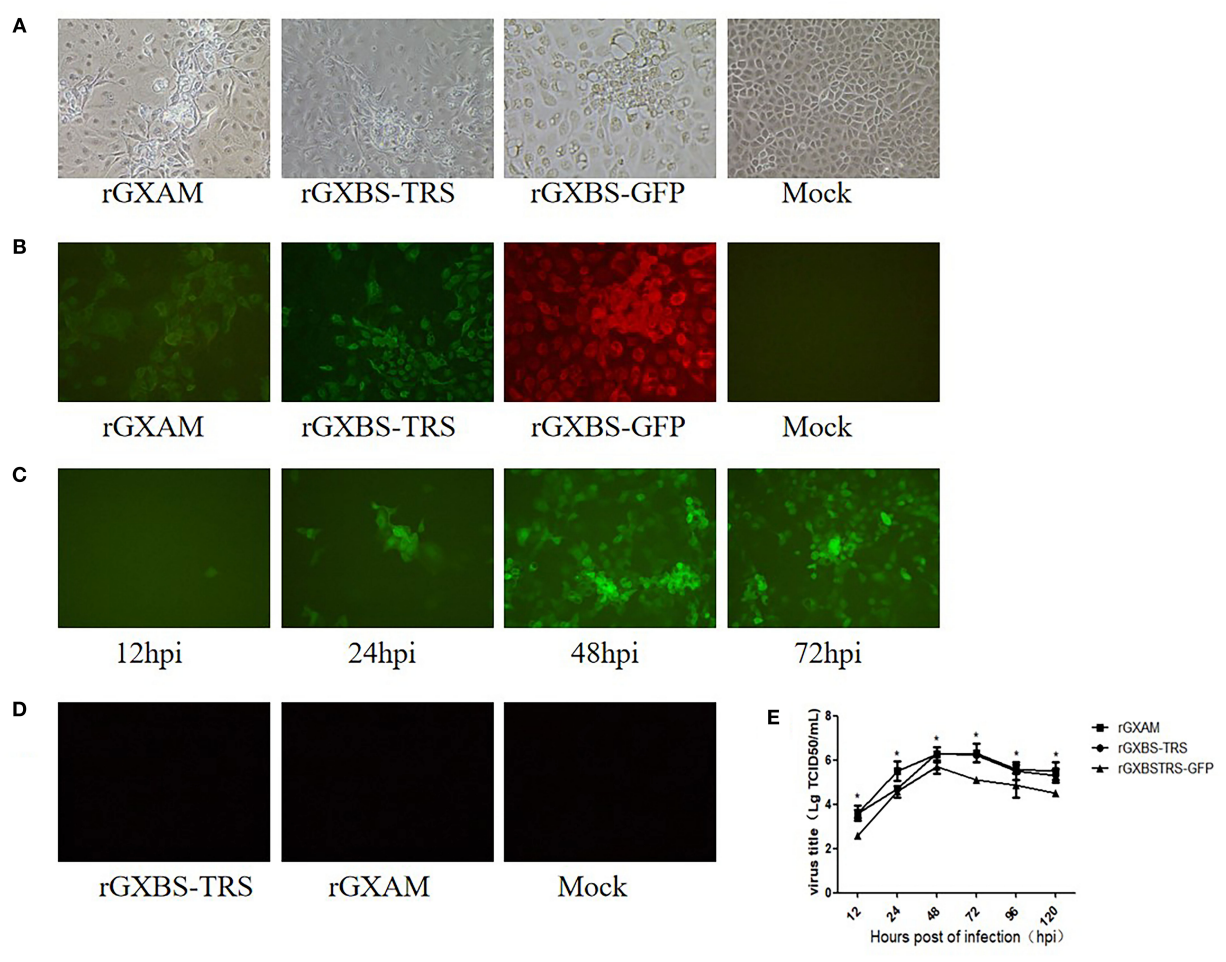
Characterization of the PRRSV virus expressing GFP inserted between ORF1 and ORF2. **(A)** Cytopathogenic effect of recombinant viruses and parental PRRSV infected MARC-145 cells. Mock infected and recombinant virus infected MARC-145 cells were observed 72 h post-transfection. **(B)** IFA of N protein expression in MARC145 cells infected with recombinant viruses and parental PRRSV. The infected MARC-145 cells were fixed and stained using a monoclonal antibody against the PRRSV N protein and anti-mouse secondary antibody labeled with Alexa Fluor 568. Images were taken at ×200 magnification. **(C)** MARC-145 cells were seeded in 6-well plates and infected with r12BSTRSGFP at a MOI of 0.1. At different times after infection, live cells were imaged with a fluorescence microscope using a 20x objective. **(D)** MARC-145 cells were seeded in 6-well plates and infected with rGXBS-TRS or rGXAM. At 72 h after infection, live cells were imaged with a fluorescence microscope. **(E)** Comparison of the growth kinetics of recombinant and parental viruses. The recombinant and the parental viruses were used to infect monolayers of MARC-cells. Subsequently, 200 μL of the supernatant was collected at the time indicated and stored at −70°C. The viral titers were determined as TCID_50_ and the values obtained were the means of using three independent experiments. Virus titers are expressed as mean ± SEM. *Indicates significant differences between the recombinant viruses and the parental virus.

CPE was generated in MARC-145 cells infected with supernatants of pGXBS-TRS, pGXBSTRS-GFP or pGXAM transfected BHK-21 cells (Figure 3A). PRRSV N protein expression was evident in these MARC-145 cells as detected by IFA (Figure 3B). GFP positive cells were observed in rGXBSTRS-GFP infected cells as early as 12 hpi. There were relatively more GFP positive cells at 48 and 72 hpi which indicated the spread of virus infection as infection progressed (Figure 3C). As expected, rGXBS-TRS, rGXAM and mock-infected cells did not show any green fluorescence (Figure 3D). To further characterize the recombinant viruses, their multi-step growth kinetics were determined using MARC-145 cells. The results showed that parental and recombinant viruses displayed similar growth behavior. However, the overall yield of recombinant virus (rBS45TRS-GFP) was less than that of the parental virus (Figure 2E). Overall, the results show that the reporter virus is replication-competent and infectious. It is suggested that pGXBS-TRS can be used as a PRRSV vector expressing foreign genes.

### Generation of a Recombinant PRRSV Stably Expressing GFP and RFP

**Figure 4:**
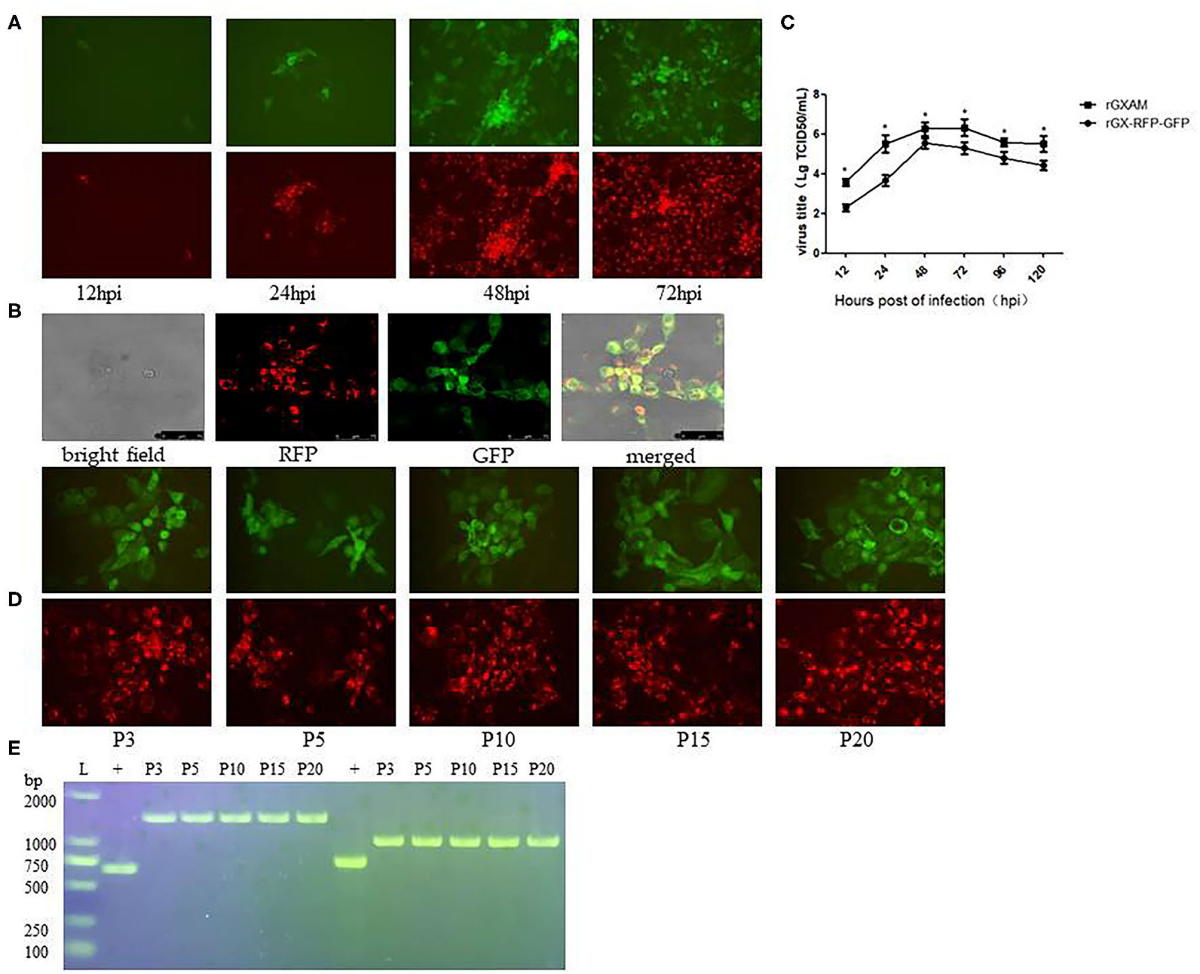
Detection of RFP and GFP protein expression of the rGX -RFP/GFP virus and the genetic stability of RFP and GFP in rGX -RFP/GFP infected MARC-145 cells. **(A)** MARC-145 cells were seeded in 6-well plates and infected with P3 rGX -RFP/GFP virus at a MOI of 0.1. At different times after infection, live cells were imaged with a fluorescence microscope using a 20x objective. **(B)** MARC-145 cells were infected with rGX -RFP/GFP virus. At 48 h post-infection, CPE generated in infected cells and corresponding fluorescence from the same field were photographed at 100 magnifications with bright field and RFP and GFP. The green and red fluorescent images were merged. **(C)** Comparison of the growth kinetics of recombinant and parental viruses. The growth of recombinant and parental viruses was compared at MOI = 0.1 on MARC-145 cells. The viral titers were determined as TCID_50_ and the values obtained were the means of three independent experiments. Virus titers are expressed as mean ± SEM. *Indicates significant differences between the recombinant viruses and the parental virus. **(D)** MARC-145 cells were infected with P3, P5, P10, P15, and P20 rGX -RFP/GFP virus. At 72 h post-infection, live cells were imaged with a fluorescence microscope. **(E)** Viral genomic RNA was extracted from clarified culture fluid rGX -RFP/GFP infected MARC-145 cells. The RFP and GFP gene inserted in P3, P5, P10, P15, and P20 rGX -RFP/GFP were amplified by RT-PCR and the products were separated on a 1% agarose gel and imaged under UV light. L, DNA ladder. + Indicates rGXAM.

To construct a recombinant PRRSV containing both RFP and GFP genes, the region covering the TRS-GFP of pGXBSTRS-GFP was replaced with the corresponding region of pGX-NSP2-RFP. The resulting construct was designated pGX-RFP-GFP. The recombinant PRRSV containing the RFP and GFP genes was recovered in MARC-145 cells inoculated with the supernatant of BHK-21 cells transfected with pGX-RFP-GFP. The RFP and GFP-positive cells were observed by fluorescence microscopy. Figure 4A shows both GFP and RFP expression was observed at each indicated time point and more positive cells were observed at 48 and 72 hpi, suggesting that the virus spread as infection progressed. The co-expression of the GFP and RFP in MARC-145 cells inoculated with rGX-RFP-GFP was examined by confocal fluorescence microscopy. After merging both RFP and GFP images, both GFP and RFP positive cells were observed and co-localized in MARC-145 cells infected with rGX-RFP-GFP (Figure 4B). The multi-step growth kinetics were determined in virus infected MARC-145 cells. The growth kinetics of rGX-RFP-GFP were similar to those of the parental virus. However, the peak titer of rGX-RFP-GFP was almost 10-fold lower than that of the original virus. Overall, the results showed that the reporter virus was infectious and genetically stable (Figure 4C).

The recombinant virus, rGX-RFP-GFP, was passaged in MARC-145 cells for up to 20 times. To determine whether the deleted or inserted sequences were genetically stable in the genomes of the recombinant viruses, the P3, P5, P10, P15, and P20 rGX-RFP-GFP were used to inoculate MARC-145 cells. RFP and GFP expression in rGX-RFP-GFP infected cells were imaged at 72 hpi. The cDNA fragments containing the targeted regions of viral genomes from P3, P5, P10, P15, and P20 rGX-RFP-GFP were amplified and then sequenced. The results showed that the majority of the MARC-145 cells infected with the P3, P5, P10, P15, and P20 rGX-RFP-GFP showed red and green fluorescence at 72 hpi (Figure 4D). In parallel with the fluorescent image results, the expected lengths of RFP and GFP fragments inserted into the genomes of different passaged viruses were detected as PCR products on agarose gels (Figure 4E). Sequence analysis showed that the recombinant viruses exhibited the introduced genes and restriction enzyme sites in the targeted regions (Supplementary Figures 1, 2). The results demonstrated that rGX-RFP-GFP is genetically stable during at least 20 serial passages in MARC-145 cells.

## Discussion

The nsp2 is the most highly heterogeneous proteins in PRRSV. Natural amino acids deletions and insertions in the HVR of nsp2 have occurred previously and these result in genome size differences amongst the PRRSV strains (15, 16). Interestingly, spontaneous amino acid deletions in nsp2 have also occurred during serial passages of PRRSV in MARC-145 cells (20, 23, 35). Some studies also showed that engineered deletion of a long coding region in the HVR of nsp2 is dispensable for virus viability (21–23). In the present study, we constructed a recombinant PRRSV clone harboring a 465-nucleotide deletion in the HVR of nsp2 that had previously occurred naturally in a PRRSV strain isolated in our laboratory (Genbank No. MN816496). As expected, the rescued virus carrying the 465-nucleotide deletion had similar growth kinetics to the parental virus indicating that this 465-nucleotide sequence in the nsp2 coding region is not essential for virus viability. The nsp2 gene HVR was also used for insertion of the GFP gene (23, 24, 36), but most recombinant viruses carrying the GFP gene fused with nsp2 were genetic unstable and lost the green fluorescence during the process of passages of the mutant virus in MARC-145 cells (23, 24, 36). In this study, we addressed this issue by inserting the RFP gene in the 465-nucleotide deleted region in the HVR of nsp2. We chose this 456-nucleotide deleted region for foreign gene insertion because this large amino acid deletion region had previously occurred naturally in a PRRSV strain and this region was supposed to tolerate the insertion of a foreign gene. As expected, the recombinant virus carrying the RFP fused with the HVR of nsp2 maintained its stability and showed RFP fluorescence after at least 20 serial passages in MARC-145 cells. It is suggested that this specific 456-nucleotide sequence deleted region in the HVR of nsp2 may be an ideal site for stably expressing foreign genes.

PRRSV vectors expressing foreign genes from an expression cassette that were inserted between ORF1b and ORF2a have been investigated systematically (27–32). The recombinant PRRSVs expressing foreign genes between ORF1 and 2a have proven stability and still possess the biological activities of the foreign genes both *in vitro* and *in vivo* (27–32). In the present study, we used the same strategy to test the ability of a cell adapted attenuated PRRSV strain to express GFP from an independent transcription unit. A GFP-expressing PRRSV showed genetic stability and the biological activity of GFP during serial passages in MARC-145 cells. It is suggested that the recombinant clone can be used as a PRRSV vector for the expression of foreign genes.

Several PRRSV based recombinant viruses only expressing a foreign gene through either an internal insertion in the HVR of nsp2 and expression cassette have attempted to be used as vaccine candidates (30, 32, 36). The levels of the host immune response to the targeted immunogens and immune protection against the targeted pathogens were seen to vary due to the expression efficiency and antigenicity of the foreign antigens expressed by the recombinant viruses (25, 27, 36). The deficient immune response against the interested pathogens conferred by these recombinant PRRSVs demonstrated that the expression level from the vector or a single antigenic gene from the pathogen may not be adequate. In the present study, for the first time, we combined the internal insertion in nsp2 and independent transcription unit methods to generate a recombinant PRRSV expressing both the RFP and GFP genes. The recombinant virus (rGX-RFP/GFP) obtained showed similar growth kinetics but the infected cells produced ~1 log less recombinant virus compared to those infected with the parental strain. A GFP/RFP-expressing PRRSV showed genetic stability and the biological activities of GFP and RFP during serial passages in MARC-145 cells. The results of our study suggest that it is possible to express two or more potential useful immunogens from a specific pathogen simultaneously from a single PRRSV vector to stimulate a stronger immune response in the host animal and therefore enhance its protective efficacy. Furthermore, the expression of two or more immunogens from different pathogens in a single PRRSV vector may achieve immune protection against challenges from several different pathogens.

## Data Availability Statement

The nucleotide sequence generated in present study were submitted to the NCBI GenBank with accession number MN816496.

## Author Contributions

ZW: conceptualization, project administration, writing—review, and editing. YW and WHe: data curation. TR: formal analysis. KO, YC, and WHu: investigation. HW and XX: methodology. All authors contributed to the article and approved the submitted version.

## Conflict of Interest

The authors declare that the research was conducted in the absence of any commercial or financial relationships that could be construed as a potential conflict of interest.
