# Supplementary material for: Generation of a Recombinant Porcine Reproductive and Respiratory Syndrome Virus Stably Expressing Two Marker Genes
Source: Front Vet Sci. 2020 Oct 22;7:548282. doi: 10.3389/fvets.2020.548282 (PMC7641969; doi:10.3389/fvets.2020.548282)
Supplement: Supplementary file 1 [file Table_1.DOCX]

Supplementary Table 1. Primers used in this study.

| Primers | Sequences(5＇-3＇) | Note |
| --- | --- | --- |
| JX451F | TGCACGAATGACTAGTGAAAACC^1^ | Primers for amplification of P2 flagment |
| JX6463R | GGGCGGCCGCGAAGGCATAGGTGCTTAAGTT |  |
| Nsp2 D155 BstZ17I F | AATGACACCAACCCTGCAGTATACAAACCTGCAGGAGCGCCCTCCAAGGGAGAA | Primers for deletion of 155 amino acids and insertion of Bstz17 I and Sbf I sites |
| Nsp2 D155 SbfI R | TTCTCCCTTGGAGGGCGCTCCTGCAGGTTTGTATACTGCAGGGTTGGTGTCATT |  |
| GX2530F | TTCCCCGCCGAGCGCTGCGGACGCTTC |  |
| GX3921R | CACACGCCGAGAAGACCCAGAAAATA |  |
| RFP BstZ17I F | GACACCAACCCTGCAGTATACATGGTGAGCAAGGGCGAGG | Primers for the amplification of RFP gene |
| RFP SbfI R | TCCCTTGGAGGGCGCTCCTGCAGGCTAGTTTCCGGACTTGTACAGCTCG |  |
| GF11706 | GCGTTTCGGGCGCGCCAGAAAGG | Primers for insertion of Bstb I and Sbf I sites |
| 12BSR | AGGCTTTGCATAGACCCCATTTCATCCTGCAGGTACC TTCGAATTCAATTCAGGCCTAAAGTTGTTC |  |
| 12BSF | GAACCAACTTAGGCCTGAATTGAATTCGAAGGTACC TGCAGGATGAAATGGGTCTATGCAAAGCCT |  |
| JX14402Mlu I R | ACGCCGGACGACAAACGCGTGGTTATCA |  |
| Sbf I-TRS-F | ACGCCTGCAGG**ATGGTTCCGCGGCAACCCCTTTAACCAGAGTTTCAGCGGAACAATATGAA**ATGGGGTCTATGCAAAGCCTCTTTAACAAGATTG^2^ | Primers for introduction of the TRS sequence |
| GFP-Bstb I-F | GAGTTCGAAATGCCCGCCATGAAGATC | Primers for the amplification of GFP gene |
| GFP-Sbf I-R | ATATCCTGCAGGCTAGGCGAATGCGATCGGGGTCTTGAA |  |
| NSP2-F | TGAGCCTCTGGATTTGTC | Primers for detection RFP gene |
| NSP2-R | ACATCCACGCCGTCTGAA |  |
| 12BS-F | ATGATGCGTTTCGGGCGCGCCAG | Primers for detection GFP gene |
| 12BS-R | CATAGTGTATAATAGTACTTTGAATC |  |

^1^The introduced restriction sites are underlined. ^2^The bold letters indicate inserted TRS core and flanking sequences.
