# Supplementary figures and images for: Generation of a Recombinant Porcine Reproductive and Respiratory Syndrome Virus Stably Expressing Two Marker Genes
Source: Front Vet Sci. 2020 Oct 22;7:548282. doi: 10.3389/fvets.2020.548282 (PMC7641969; doi:10.3389/fvets.2020.548282)

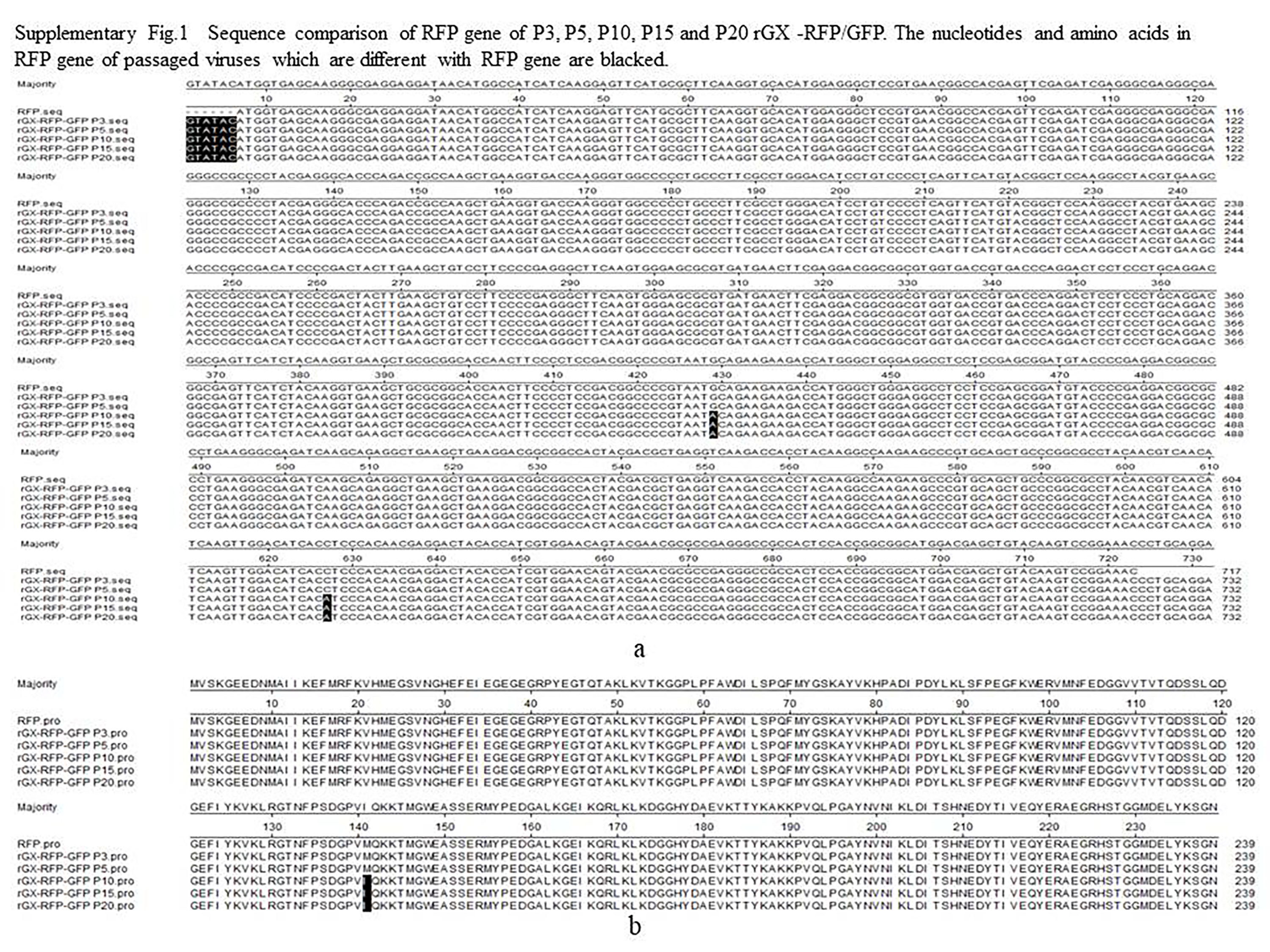

Supplement: Supplementary file 2 [file Image_1.JPEG]

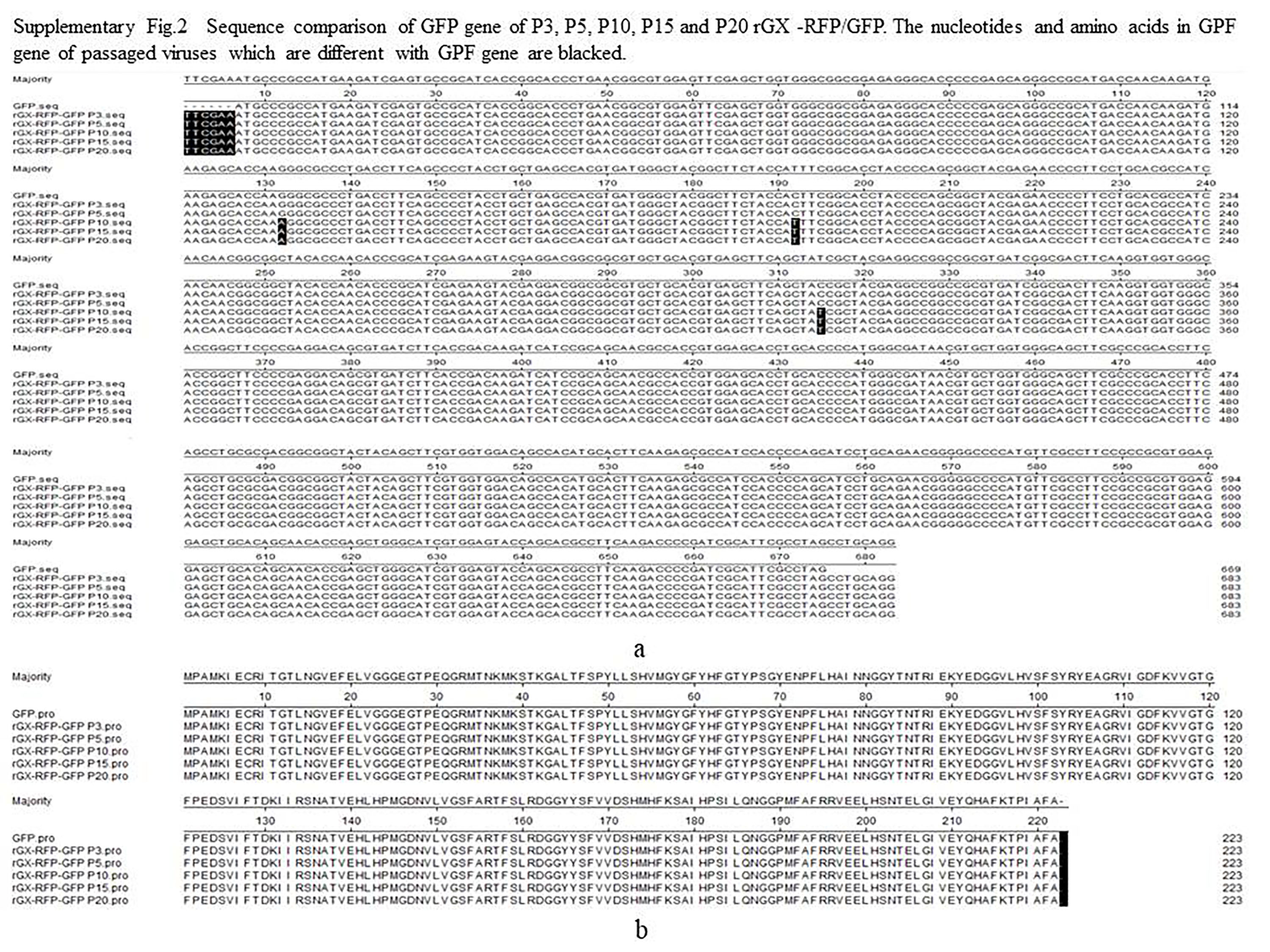

Supplement: Supplementary file 3 [file Image_2.JPEG]
